# Reemergence of *Mycobacterium chimaera* in Heater–Cooler Units despite Intensified Cleaning and Disinfection Protocol

**DOI:** 10.3201/eid2210.160925

**Published:** 2016-10

**Authors:** Peter W. Schreiber, Stefan P. Kuster, Barbara Hasse, Cornelia Bayard, Christian Rüegg, Philipp Kohler, Peter M. Keller, Guido V. Bloemberg, Francesco Maisano, Dominique Bettex, Maximilian Halbe, Rami Sommerstein, Hugo Sax

**Affiliations:** University Hospital Zurich, Zurich, Switzerland (P.W. Schreiber, S.P. Kuster, B. Hasse, C. Bayard, C. Rüegg, P. Kohler, F. Maisano, D. Bettex, M. Halbe, H. Sax);; University of Zurich, Zurich (P.W. Schreiber, S.P. Kuster, B. Hasse, C. Bayard, C. Rüegg, P. Kohler, F. Maisano, D. Bettex, H. Sax);; National Centre for Mycobacteria at University of Zurich, Zurich (P.M. Keller); Institute of Medical Microbiology at University of Zurich, Zurich (P.M. Keller, G.V. Bloemberg);; University Hospital, Bern, Switzerland (R. Sommerstein);; University of Bern, Bern (R. Sommerstein)

**Keywords:** *Mycobacterium chimaera*, bacteria, hospital infections, hospital equipment, disinfection, nontuberculous mycobacterial infections, heater–cooler units, tuberculosis and other mycobacteria, nosocomial infections

## Abstract

Invasive *Mycobacterium chimaera* infections after open-heart surgery have been reported internationally. These devastating infections result from aerosols generated by contaminated heater–cooler units used with extracorporeal circulation during surgery. Despite intensified cleaning and disinfection, surveillance samples from factory-new units acquired during 2014 grew nontuberculous mycobacteria after a median of 174 days.

*Mycobacterium chimaera* is an emerging pathogen causing disastrous infections of heart valve prostheses, vascular grafts, and disseminated infections after open-heart surgery (*1*,*2*). Growing evidence supports airborne transmission resulting from aerosolization of *M. chimaera* from contaminated water tanks of heater–cooler units (HCUs) that are used with extracorporeal circulation during surgery (*3*,*4*). HCUs were previously associated with surgical site infections caused by nontuberculous mycobacteria (NTM) (*5*). We describe the colonization dynamics of factory-new HCUs with NTM during regular use.

## The Study

Identification of *M. chimaera* infection in 6 patients prompted an outbreak investigation at the University Hospital Zurich, a 900-bed tertiary-care hospital in Zurich, Switzerland, that performs ≈700 open-heart surgeries that use extracorporeal circulation per year. The investigation included microbiologic sampling of HCUs for NTM (*3*). Surveillance cultures of HCU water from the cardioplegia and patient circuits and airflow samples from running HCUs at ≈2–3 m distance were gathered in monthly intervals. Failure to eradicate *M. chimaera* and other NTM from older HCUs prompted the acquisition of 5 factory-new HCUs (model 3T; Sorin [now LivaNova, London, UK]) during 2014: 2 in January, 1 in April, and 2 in September. 

Mycobacterial cultures were performed according to standard methods by using the mycobacteria growth indicator tube system (MGIT 960; Becton Dickinson, Sparks, MD, USA) or Middlebrook 7H11 agar plates (BD Difco Mycobacteria 7H11 Agar; Becton Dickinson) that were incubated at 37°C for 7 wks or until positive. Air specimens were gathered with a microbiologic air sampler (MAS-100 NT, MBV, Stäfa, Switzerland) running for 2.5 min at a rate of 100 L/min by using Middlebrook 7H11 agar plates. Mycobacterial species were identified by 16S rRNA gene sequencing, as described (*6*).

**Figure 1:**
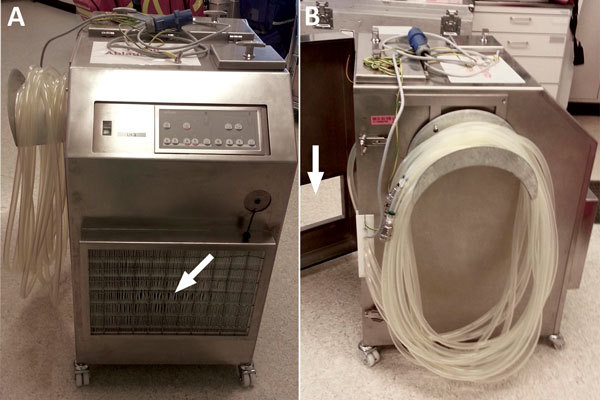
Custom-made stainless steel housing for heater–cooler units (model 3T; Sorin [now LivaNova, London, UK]) used at the University Hospital Zurich, Zurich, Switzerland. A) Front view shows the fine dust filter F7 over the air inlet (arrow). B) Side view shows the half-open back door with the rectangular opening (arrow), through which a duct connects the housing to the operating room ventilation exit. The negative pressure of the operating room ventilation system generates the necessary airflow.

Before mid-April 2014, HCUs were serviced according to the manufacturer’s recommendations. Before first use and every 3 months thereafter, a disinfection cycle was performed by adding 200 mL of 3% sodium hypochlorite (Maranon H; Ecolab, Northwich, UK) to the HCU water tanks filled with filtered tap water (Pall-Aquasafe Water Filter AQ14F1S; Pall, Portsmouth, UK) to a final concentration ≈0.045% sodium hypochlorite in the water tank and circuits. Water was changed every 14 days; 100 mL of 3% hydrogen peroxide was added to the initial water filling (final concentration ≈0.02% hydrogen peroxide in water tank and circuits); an additional 50 mL of 3% hydrogen peroxide was added every 5 days (*7*). In mid-April 2014, an intensified in-house cleaning and disinfection procedure was implemented, consisting of daily water changes with filtered tap water (Pall) and additions of 100 mL of 3% hydrogen peroxide combined with biweekly disinfection using sodium hypochlorite (Maranon H). In February 2015, with lack of availability of 3% sodium hypochlorite and in line with the manufacturer’s recommendations, the disinfection solution was changed to a combination of peracetic acid and hydrogen peroxide (an additional 450 mL Puristeril 340; Fresenius Medical Care, Hamburg, Germany) in filled water tanks every 2 weeks. Also, stainless steel housings were custom built around the HCUs to ensure strict air separation between the exhaust air of the HCUs and the operating room air (Figure 1). These housings were directly connected to the operating room exhaust conduit.

**Figure 2:**
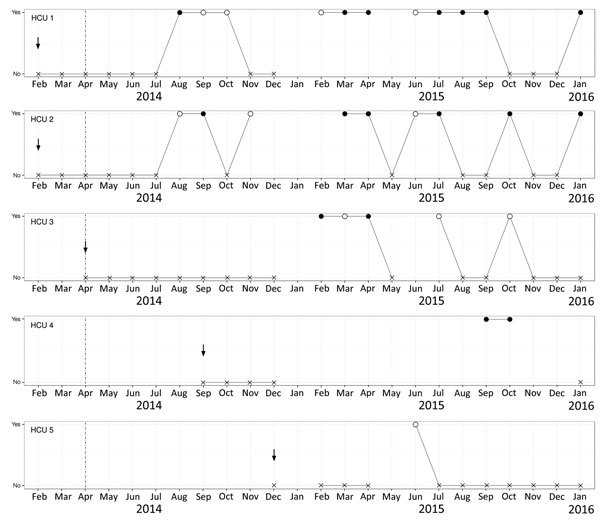
Results of heater–cooler unit (HCU) water surveillance cultures by year and month, University Hospital Zurich, Zurich, Switzerland. The dashed vertical line shows the date of implementation of the intensified protocol (i.e., mid-April 2014). The vertical arrows indicate the start of use of each factory-new HCU in the operating room. HCU 3, HCU 4, and HCU 5 were serviced with the intensified in-house maintenance from the time of delivery. HCU 4 was sent for repair at the manufacturer during December 2014–September 2015. Results of mycobacterial cultures are shown for each HCU. No indicates a negative culture for nontuberculous mycobacteria (NTM), indicated by X. Yes indicates a positive culture for NTM. Filled circles indicate *Mycobacterium chimaera*; empty circles indicate NTM other than *M. chimaera*. As per date indicated, cultures were reported as positive if ≥1 water sample (1 sample from cardioplegia and 1 sample from patient circuit were gathered at each date) grew NTM. For missing data points (i.e., no negative or positive results shown), mycobacterial cultures could not be tested because of bacterial overgrowth or lack of availability of the HCU.

A total of 134 water samples were obtained from the study HCUs, 127 after implementation of the intensified protocol. The first water sample tested positive for *M. chimaera* in August 2014, originating from a study HCU introduced 7 months earlier (Figure 2). Of all samples, 90 (67.2%) remained sterile for NTM; 6 (4.5%) were contaminated by bacterial overgrowth; and 38 (28.4%) yielded NTM. Of these NTM, 22 (57.9%) were *M. chimaera*; 12 (31.6%) were *M. gordonae*; 1 (2.6%) was *M. chelonae*; 1 (2.6%) was *M. paragordonae*; and 2 (5.3%) were a combination of *M. chimaera* and *M. gordonae*. NTM were found in both cardioplegia and patient HCU water circuits (Table).

**Table:** Microbiology test results for heater–cooler unit water samples from University Hospital Zurich, Zurich, Switzerland*

Type of circuit	No. samples	Microbiology results, no. (%) samples		
		Any NTM growth	*Mycobacterium chimaera*	NTM other than *M. chimaera*
Cardioplegia†	48	14 (29.2)	10 (20.8)‡	5 (10.4)‡
Patient†	49	19 (38.8)	12 (24.5)‡	8 (16.3)‡
Circuit not specified	37§	5 (13.5)	2 (5.4)	3 (8.1)

*NTM, nontuberculous mycobacteria. †No statistically significant different results for water samples from cardioplegia and patient circuit (Fisher exact test, p = 0.554). ‡One culture had growth of both *M. chimaera* and *M. gordonae*. §Six (16.2%) samples had bacterial overgrowth.

Of 91 air samples, 90 (98.9%) had no mycobacterial growth. One sample grew *M. chelonae*, although no mycobacterium was detected simultaneously in the corresponding HCU water.

NTM growth was recorded after a median of 174 (range 158–358) days in HCU water samples. One of 5 HCUs remained permanently without growth of *M. chimaera*; 4 grew *M. chimaera* after a median of 250 (range 158–358) days.

## Conclusions

HCUs seem to provide favorable environmental conditions for growth of NTM, in particular *M. chimaera*. An intensified cleaning and disinfection protocol failed to prevent growth of NTM entirely but succeeded in preventing detectable aerosolization of *M. chimaera*.

The contamination status of HCUs seems to be influenced by the intensity of maintenance, especially frequency of water changes. This hypothesis led to the development of the in-house maintenance protocol. The consistently negative air cultures for *M. chimaera* and the only intermittently positive water cultures support the benefits of our intensified protocol.

Our study design did not elucidate the origin of *M. chimaera* and other NTM in HCUs. The HCUs might have been already contaminated at time of delivery in a concentration below the detection threshold of mycobacterial cultures. A recent investigation confirmed the presence of environmental mycobacteria, including *M. chimaera*, in factory-new HCUs (*8*,*9*). In our study, 1 HCU (HCU 4, Figure 2) tested positive for *M. chimaera* for the first time after being returned from repair at the manufacturer. Contamination from tap water is unlikely because the study HCUs used only filtered water.

Previous studies indicated the durability of mycobacteria against several disinfectants, likely because of the organisms’ complex cell wall (*10*). *M. avium* complex isolates were shown to have a high level of resistance against chlorine when grown in water (*11*). Corrosion, certain material characteristics, and dead-end spaces can favor biofilm formation and mycobacterial growth. Killing of NTM with heat may be promising; older studies reported a high efficacy with temperature exposure at 70°C (*10*,*12*). A recent report indicated complete suppression of *M. chimaera* in HCUs by intensified maintenance after a complex decontamination regimen, including replacement of plastic tubing; however, follow-up was limited to 3 months (*13*). Prolonged testing seems necessary for excluding presence of *M. chimaera* or other NTM.

Our report has limitations. First, some study HCUs were temporarily maintained according to the manufacturer’s standard before the hospital adopted the intensified protocol. Second, we did not include a control HCU with ongoing maintenance according to the manufacturer’s recommendations. Third, the detection threshold of *M. chimaera* in water and air cultures remains to be identified. More sensitive culture methods might have produced different results.

Our findings challenge the effectiveness of the HCU manufacturer’s maintenance recommendations, which were recently changed to disinfection with sodium hypochlorite before first use and every 14 days and water changes with all-bacteria–filtered tap water plus 150 mL 3% hydrogen peroxide every 7 days (*14*). To ensure patient safety until safe HCU technology is available, strict separation of the operating room and HCU air volumes is necessary. This separation can be achieved in several ways. One approach is placing the HCU outside the operating room. Nevertheless, with this measure, the airflow must be restrained from diffusion back into the OR (*15*). Because the maximum allowed length of water circuit tubing and the architectural layout prohibited this solution at our hospital, we produced airtight housings for the HCUs; however, this solution has less flexible placement of the HCUs within the OR. We continue both the in-house maintenance protocol and regular microbiologic surveillance.
